# P-1108. Efficacy and Safety of Investigational Therapeutic Microbiome SER-109 in Recurrent Clostridioides difficile Infection: A Systematic Review

**DOI:** 10.1093/ofid/ofae631.1296

**Published:** 2025-01-29

**Authors:** Stephanie P Fabara, Akankcha Alok, Harsimran Kalsi, Mohamed Bakhit, Sanil Thomas

**Affiliations:** UCF/HCA FL North Florida Hospital, Gainesville, Florida; University of Central Florida, Gainesville, Florida; UCF/HCA FL North Florida Hospital, Gainesville, Florida; University at Buffalo, Buffalo, New York; HCA Florida North Florida Hospital, Gainesville, Florida

## Abstract

**Background:**

Current therapies for recurrent *Clostridioides difficile* infection (CDI) often fail to address the disrupted microbiome contributing to *C. difficile* spore germination and subsequent toxin production. SER-109, an investigational therapeutic microbiome, is composed of purified Firmicutes spores and shows promise in treating recurrent CDI. Our aim was to evaluate the efficacy, safety and recurrence rate of CDI following administration of oral SER-109.

**Figure 1.**
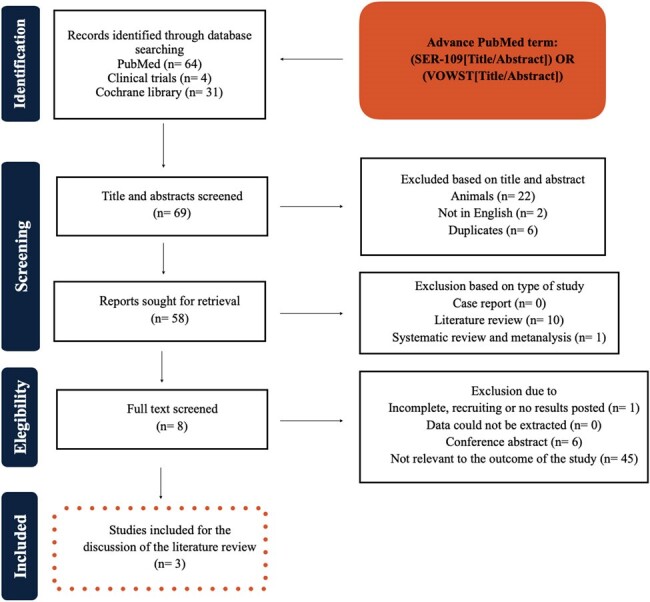
Selection of studies using a PRISMA flow chart.

**Methods:**

We systematically reviewed available literature on PUBMED using the Preferred Reporting Items for Systematic Reviews and Meta-Analysis (PRISMA) for clinical trials involving SER-109 (Figure 1). We found three clinical trials that met our inclusion criteria and primary efficacy endpoint criteria (Table 1). For assessing bias, we used the Cochrane Collaboration's tool risk assessment of the clinical trials (Figure 2).

**Table 1.**
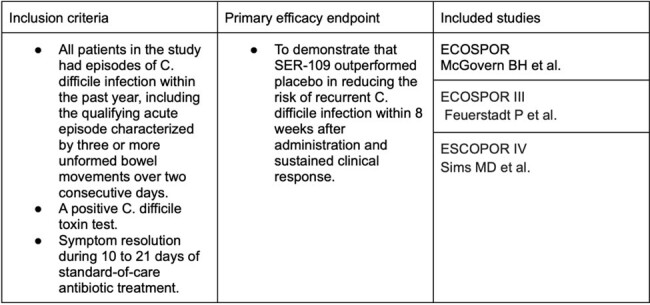
Inclusion criteria and primary efficacy endpoint

**Results:**

Oral SER-109 was well tolerated with the rate of recurrent CDI remaining low, regardless of the number of prior recurrences or patient demographics. The diagnostic approach did not significantly impact the beneficial effect of SER-109. In patients with symptom resolution of C. difficile infection after treatment with standard-of-care antibiotics, oral administration of SER-109 was superior to placebo in reducing the risk of recurrent infection. The observed safety profile of SER-109 was mild and similar to that of placebo. Our findings are summarized in Table 2.

**Figure 2.**
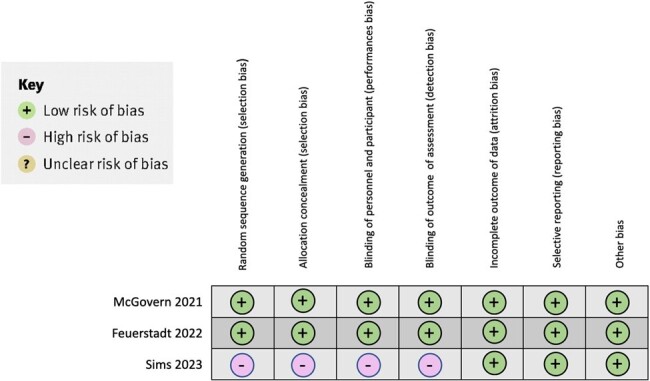
Bias analysis of the clinical trials in the systematic review.

**Conclusion:**

SER-109 holds promise as a safe and effective treatment for recurrent CDI. Further research is warranted to confirm its long-term efficacy and explore potential mechanisms underlying its impact on the disrupted gut microbiome.

**Table 2.**
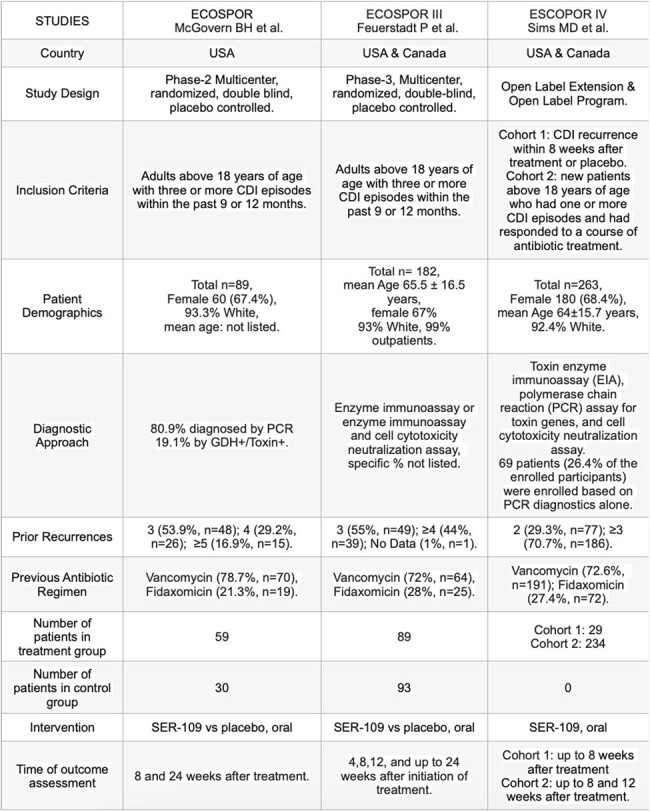
Characteristics and outcomes of selected studies.

**Disclosures:**

**All Authors**: No reported disclosures

